# Curcumin Veto the Effects of Osteopontin (OPN) Specific Inhibitor on Leukemic Stem Cell Colony Forming Potential via Promotion of OPN Overexpression

**Published:** 2016-07-01

**Authors:** Saeed Mohammadi, Seyed H. Ghaffari, Mojgan Shaiegan, Mahin Nikogoftar Zarif, Mohsen Nikbakht, Kamran Alimoghaddam, Ardeshir Ghavamzadeh

**Affiliations:** 1 Iranian Blood Transfusion Research Center, High Institute for Education and Research in Transfusion Medicine, Tehran, Iran; 2 Hematology-Oncology and Stem Cell Transplantation Research Center, Tehran University of Medical Sciences, Tehran, Iran

**Keywords:** Curcumin, Osteopontin, Leukemic stem cell, Colony forming potential, SiRNA

## Abstract

**Background:** Acute myeloid leukemia (AML) is an immunophenotypically heterogeneous malignant disease, in which CD34 positivity is associated with poor prognosis. Osteopontin (OPN) plays different roles in physiologic and pathologic conditions like: survival, metastasis and cell protection from cytotoxic and apoptotic stimuli. Due to anti-apoptotic effect of OPN in normal and malignant cells, silencing of OPN leads to elevation of sensitivity towards chemotherapeutic agents and attenuates cancer cells migration and invasion. Therefore, the aim of this study was to evaluate OPN roles in modulating curcumin-mediated growth inhibitory on leukemic stem cells (LSCs) colony forming potential and survival in AML cell lines and primary CD34+/CD38- bone marrow-derived AML cells.

**Materials and Methods:** Primary human CD34+/CD38- cells were isolated from bone marrow mononuclear cells of 10 AML patients at initial state of diagnosis, using a CD34 Multi sort kit. The growth inhibitory effects of curcumin (CUR) were evaluated by MTT and colony-formation assays. Apoptosis was analyzed by 7AAD assay in CD34+ KG-1, U937 cell lines and primary isolated cells. Short interfering RNA (siRNA) against OPN was used for OPN silencing in both cell lines and primary AML cells. Then, transfected cells were incubated with/without curcumin. The change in OPN gene expression was examined by Real-time PCR.

**Results:** CUR inhibited proliferation and induced apoptosis in both KG-1 and U937 cells and also primary isolated AML cells. OPN silencing by siRNA increased the susceptibility of KG-1, U937 and primary CD34+/CD38- AML cells to apoptosis. Moreover, soft agar colony assays revealed that silencing of OPN with siRNA significantly decreased colony numbers in LSCs compared with the non-targeting group. Furthermore, CD34+/CD38- populations as a main LSCs compartment through OPN overexpression towards CUR treatment might be nullified the inhibitory effects of OPN siRNA on their survival and colony forming potential.

**Conclusion:** Taken together, our results suggested that knockdown of OPN using OPN specific siRNA significantly decreased colony numbers in LSCs and this effect might be vetoed by LSCs via induction of OPN overexpressionin combination of CUR and siRNA.

## Introduction

 Acute myeloid leukemia (AML) is one of the most common leukemias in adults. AML is characterized by an accumulation of undifferentiated and heterogeneous populations of cells.^        1 ^ Overlay, any treatment that reduces the tumor burden less than 10^9^ will result in a clinical complete remission (CR). Even with high-dose chemotherapy, only 30%-40% of AML patients survive, which is due mainly to relapse of the disease.^   2 ^ Hence, it is clear that there is a rare subset of malignant cells that are not effectively eradicated by current treatment regiments.^   3 ^ LSCs may justify this failure of the CR.^        4 ^ The phenotype of LSCs may be somehow variable from patient to patient and even, in some cases, more than one phenotypically distinct subpopulation may possess LSC activity. However, It is suggested that they mainly reside within the CD34+/CD38- compartment of leukemic clone and CD34+‏ CD38‏+ and CD34- populations are other fractions of LSCs.^        5 ^^,^^        6 ^

Osteopontin (OPN), encoded by a single gene on chromosome 4q13, 7^,^^8^  is secreted by many cell types such as lymphocytes, osteoclasts, endothelial cells and tumoral cells.^        9 ^ OPN is elevated in stromal cells-mediated tumor microenvironment^        10 ^ and plays different roles in physiologic and pathologic conditions like: invasion, metastasis, survival, angiogenesis, tumorigenesis, tumor growth,^11^^,^^12^ regulation of bone hemostasis and cell protection from cytotoxic and apoptotic stimuli.^        13 ^ Since curative treatment of leukemia will most likely require the elimination of LSCs, new strategies need to be designed that overcome the drug resistance of these cells. In this respect, strategies that target a distinct Achilles' heel of the LSC biology appear to be particularly promising. Furthermore, most of the compounds that have been shown to successfully eradicate LSCs are known to be inhibitors of NF-kB.^        14 ^ In this regard, it seems curcumin (CUR) is a suitable choice to large extent. CUR, one of different molecules in Curcuma longa (diferuloylmethane) a member of the Zingiberacae (ginger) family, has several properties such as anti-inflammatory, antioxidant and antimicrobial activities. ^15^^,^^16^  Inhibition of cancer cell proliferation, invasion, metastasis, angiogenesis and also apoptosis induction^   17 ^ by disruption of molecular targets which inhibit the signaling pathways including NF-kB,^        18 ^ AKT/mTOR and HIF-1α molecules.^17^^,^^19^^,^^20^ Although CUR induces apoptosis in variety of AML cell lines, cytotoxic effects of CUR in LSCs remain unclear. Despite the well-defined functions for OPN in solid tumor and benign HSC biology, there is little data regarding OPN and leukemia. Recently, a few reports have been published in hematological malignancies.^21^^,^^22^ Due to anti-apoptotic effect of OPN in normal and malignant cells; ^23^^-^^25^  so, silencing of OPN that leads to elevation of sensitivity towards chemotherapeutic agents and attenuates cancer cells migration and invasion. ^26^^,^^27^  In this regard, based on the above mentioned, maybe CUR is probably considered choice. Therefore, the aim of present study was to evaluate the effect of CUR and OPN specific siRNA on LSCs survival and colony forming potential. In this study, we have demonstrated that LSCs via OPN overexpression at mRNA level towards CUR treatment might be vetoed the inhibitory effects of OPN siRNA on survival and colony forming potential.

## MATERIALS AND METHODS

**Reagents and antibodies**

Curcumin (CUR) (Sigma, St. Louis, MO) was dissolved in dimethyl sulfoxide (DMSO) (Sigma, St. Louis, MO) to provide a 100 mM stock solution and stored at -20°C. 7AA Dassay kit (BD Biosciences; San Jose, CA, USA), CD34 Multi sort, Micro Bead kit (Miltenyi biotec, Auburn, CA, USA) and 3-(4, 5-dimethylthiazole-2-yl)-2, 5-diphenyltetrazolium bromide (MTT) dye were obtained from Sigma-Aldrich (St Louis, MO, USA). CD34-RPE, CD38-FITC (BD Biosciences; San Jose, CA, USA) and Methocult semi-solid media purchased from (Stem Cell Technologies, Vancouver, BC, Canada).

**Cell lines and cell culture**

KG-1 and U937 cell lines were purchased from Pasteur institute of Iran. KG-1 cells were cultured in DMEM medium and supplemented with 20% FBS (Gibco; Invitrogen, USA), 2 mM L-glutamine, 100 units/ml penicillin and 100 µg/ml streptomycin. U937 cells were cultured in RPMI 1640 medium supplemented with 10% FBS (Gibco; Invitrogen, USA), 2 mM L-glutamine, 100 units/mL penicillin and 100 µg/mL streptomycin. Cells were incubated at 37°C in a humidified atmosphere containing 5% CO2. For CUR treatment, a relevant amount of working solution (5 mM in DMSO) of CUR was added to culture medium to attain the concentrations of 20, 40, 60, 80 µM. Control cultures were received an equivalent amount of DMSO (0.1%) during treatment with CUR.

**Cell separation, cell sorting and primary culture**

Totally, 10 bone marrow aspirations were collected from patients at initial time of diagnosis and prior to any treatment during admission in Hematology-Oncology ward, Shariati Hospital. The study was approved by Ethical committee and informed consent was obtained from all of patients recruited in the study (Ethical code: Ir.tums.horcsct. 1394.103.5).

Diagnoses were based on standard hematological protocol and classification which was according to French-American-British classification. The clinical characteristic of leukemia patients has been shown in Table 1. Bone marrow mononuclear cells were obtained by Ficoll-Hypaque density gradient centrifugation. CD34+/CD38- cells were purified from these mononuclear cells by multi sort CD34 MACS Column Technology according to manufacturer protocol. The purity of enriched CD34+/CD38- was evaluated by staining with FITC-conjugated anti-CD34 and CD38-PE. The isolated CD34+/CD38- cells were treated with CUR and cultured in RPMI 1640 medium supplemented with 10% FBS (Gibco; Invitrogen, USA), 2 mM L-glutamine, 100 units/mL penicillin and 100 µg/mL streptomycin and incubated at 37°C in 5% CO2 incubator for indicated tests. Control cultures received an equivalent amount of DMSO (0.1%) during treatment with CUR.

**MTT assay**

Micro culture Tetrazolium Test (MTT) assay was used to test the effect of CUR on viability of KG-1 and U937 cell lines. The cells at density of 5,000 cells/100 μl in 96-well culture plates (SPL Life sciences, Pocheon, Korea), were treated with desired concentration of CUR and incubated in a humidified tissue-culture chamber (37^0^C, 5% CO2) for 24-48h. MTT solution (0.5 mg/ml) was added to each well and the plate was incubated at 37 C in 5% CO2 atmosphere. DMSO was added to each well and incubated for 4h to liquefy the dye crystals. Absorbance at a wavelength of 570 nm was gauged by an ELISA plate reader (Micro plate Reader; Bio-Rad). Cell treated with 0.1% DMSO was defined as the control group.

**In vitro colony formation assay**

Treated and untreated KG-1, U937 cell lines and primary CD34+/CD38- AML cells were suspended in RPMI 1640 medium. Colony formation assay was done in Methocult semi-solid media (Stem Cell Technologies, Vancouver, BC, Canada). The cell density was 2000/mL/plate and cells were incubated at 37°C for 14 days. Routine colony counts were evaluated by inverted microscope. Accumulation of 50 cells or more were scored as one colony and collection of 3~50 cells were as one cluster. Three independent experiments were carried out.

**RNA isolation and Real-time PCR**

Tripure isolation Reagent (Roche Applied Science, Germany) was used for total RNA extraction according to the manufacturer’s instruction. The quantity of RNA samples were assessed spectrophotometrically using Nanodrop ND-1000 (Nanodrop Technologies, Wilmington, DE). Complementary DNA (cDNA) synthesis was performed by using cDNA synthesis kit (Takara Bio Inc., Otsu, Japan). Real-Time PCR was performed with Step One Plus™ ABI instrument (Apply Bio systems, USA) using 2 μl of a 2-fold diluted cDNA, 0.5 µl of each forward and reverse primers (10 pMol) and SYBR Premix Ex Taq technology (Takara Bio Inc, Otsu, Japan) in a final volume of 20 μl. HPRT mRNA expression levels were used to estimate the relative expression levels. Thermal cycling conditions included a predenaturation step for 30s at 95^0^C followed by 45 cycles including a denaturation step for 5s at 95^0^C and a combined annealing/extension step for 20s at 60^0^C. The specificity of PCR reactions was confirmed by melting curve analysis. The fold change for each mRNA in treated leukemic cell lines in comparison with untreated cells was computed by the 2^-ΔΔCT^ method.^28^ The primers and their corresponding amplicon lengths were provided in Table 2.

**Short interfering RNA (siRNA) transfection**

The effect of OPN mRNA expression and also efficacy of curcumin in induction of apoptosis was tested by use of RNA interference in both cell lines and patient samples.

**Table 1:** Characteristic of patients

**Patient**	**Age/Sex**	**FAB**	**%CD34 in BMC**	**Source**	**Cytogenetic**	**FLT3**	**WT-1 copy number**
P1	35Y/M	M1	32%	BM	46, XY [8]	Neg	905
P2	40Y/M	M0	38%	BM	46, XY [20]	Neg	1810
P3	30Y/M	M1	43%	BM	50, XY add (3) (p12), -5, +8, +21, +21, +marI [4]/51, XY, idem, +18, +marI [3]/50, XY, idem, +22 [2]/51, XY, idem, 22, marI [2]/52, xy, IDEM, +18, marI, +marII [4]/46, xy [5], 45, xx, -7 [49]/46, XX [1]	Neg	701
P4	50Y/F	M0	61%	BM	45, XX, -7 [49]/46, XX [1]	Neg	4302
P5	40Y/M	M2	77%	BM	45, XY, del (5) (q14;q34), der (15), t (15;17) (p11.1 ;q11.1), -17 [12]/46, XY [38]	Neg	3066
P6	28Y/M	M4	15%	BM	45, XY, [20]	Neg	780
P7	38Y/M	M2	40%	BM	45, XY, [20]	Neg	7331
P8	36Y/M	M2	45%	BM	46, XY, t (6;9) (p23;q3), t (9;18) (q34;q21) [20]	Neg	5080
P9	49Y/M	M4	30%	BM	46, XY, del (11q23) [10]	Neg	28911
P10	30Y/F	M2	43%	BM	46, XX, [20]	Neg	3532

**P:** Patient, **Y:** Year, **M:** Male**, F:** Female, **FAB:** French-American-British, **BMC:** Bone marrow cells, **%CD34 in BMC:** Percentage of CD34+ cells in bone marrow cells of AML patients before sorting, **FLT3:** Fms like tyrosine kinase, **WT-1:** Wilms Tumor-1

Small interfering RNA (siRNA) for OPN with a 21 base pair duplex as well as its control with the same base composition were designed against all variants of OPN gene and partial cds silencing in a random sequence (Table 3).

OPN specific as well as control siRNA were given in optimization experiments and maximal gene knockdown was attained 24h post-transfection by using siRNA at final concentration of 40 (pM). Impact of OPN gene knockdown on cell number and viability were evaluated on 96-well culture plates (SPL Life Sciences, Pocheon, Korea) for 24h after transfection. The siRNA were transfected using of Lipofectamine 2000 reagent (Invitrogen), according to the manufacturer’s instruction.

**Statistical analysis**

SPSS 18 was used to perform statistical analysis. The significance of differences between experimental variables was determined by the use of two-tailed student's test to compare between the control and the experimental groups. All experiments were performed in triplicate and results have been expressed as the mean ± standard deviation (SD). One-way analysis of variance (One-way ANOVA) was used to determine statistical significances of difference. Statistical significance were defined at *p<0.05, **p<0.01 and ***p<0.001 compared to corresponding control.

## Results

**CUR inhibits cell proliferation**

**Figure 1:**
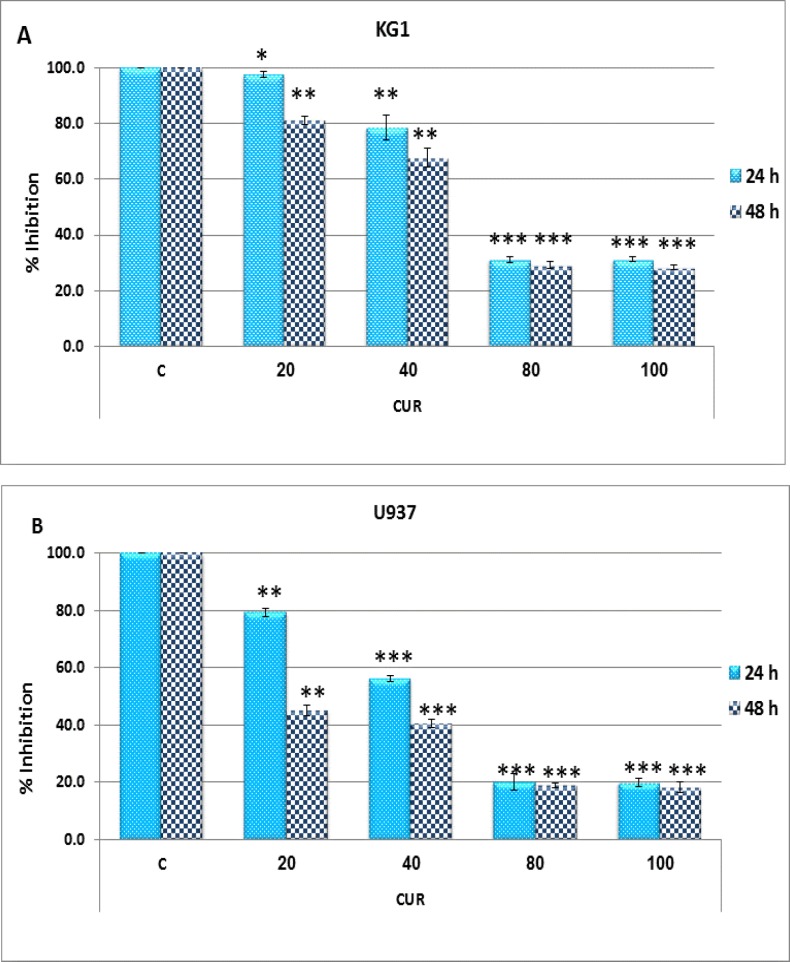
Effects of curcumin with different concentration (0-100 µM) on cell viability. Anti-growth effect of curcumin was measured by MTT assay following 24-48h exposure to KG-1 **(A)** and U937 cell lines **(B)**. Data are mean ± SD of three independent experiments. Statistical significance was defined at *p<0.05, **p<0.01 and ***p<0.001 compared to corresponding control.

**Figure 2:**
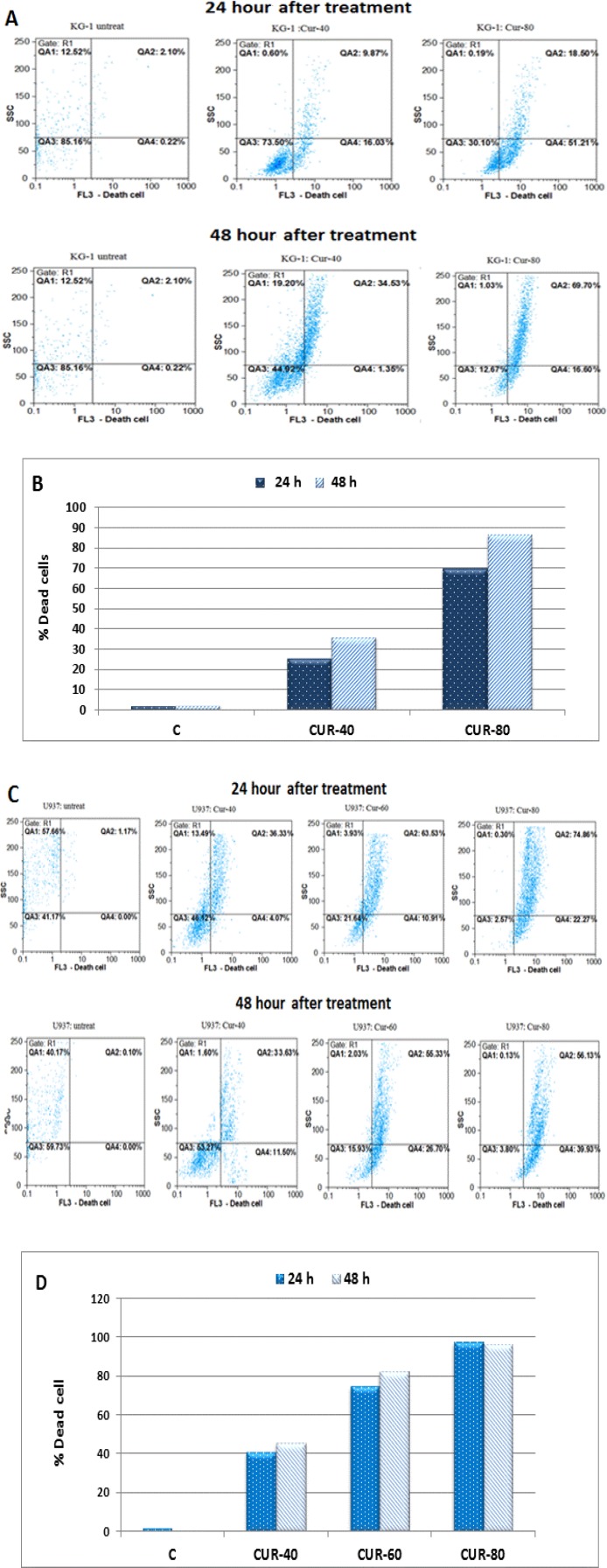
Results of 7AAD assay in KG-1 and U937 cell lines after 24-48h treatment with different concentration of curcumin (0-80 µM). **(A):** Flow cytometry histograms and **(B)** bar charts in KG-1 cell line. **(C):** Flow cytometry histograms and **(D)** bar charts in U937 cell line

Initial studies were performed to elucidate the anti-survival effects of CUR on AML cell lines. After treatment of these cell lines with different concentrations of CUR for 24-48h, growth suppressive effect were assessed by MTT methods (Figure 1: A-B). Afterward, more sensitive 7AAD method was performed to confirm result of MTT assay. The result of 7AAD and MTT showed that CUR inhibited cell proliferation with lowest IC50 value for U937 cells (40 µM) in comparison to KG-1 cell lines (80 µM). Interestingly, drug exposure time (24-48h) had no significant effect on cell growth. CUR had a significant cytotoxic effect in all tested cell lines in dose dependent manners (Figure 1: A-B and Figure 2: A-D).

**Colony assay**

**Figure 3:**
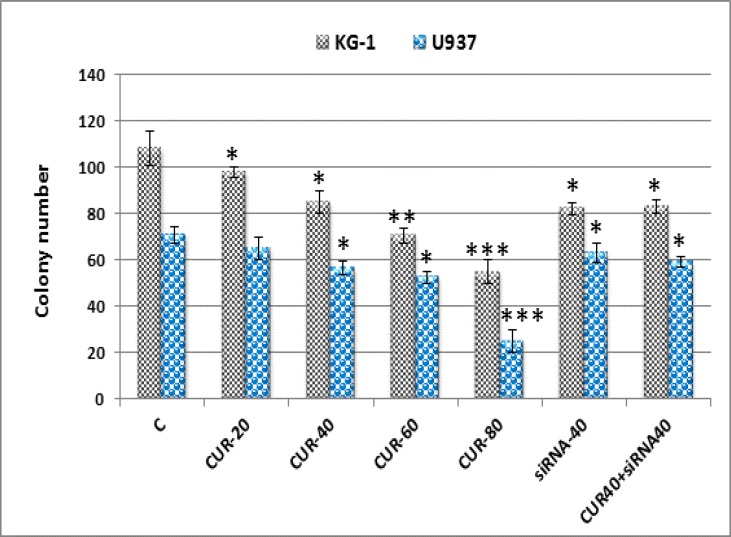
Effects of curcumin (40 µM) and siRNA (40 pM) on colony forming potential. Colony formation assay confirms great decrease in colony count by curcumin and siRNA in both cell lines. Values are given as mean ± SD of three independent experiments.

To assess the effects of CUR treatment, in vitro colony assays was used to determine whether CUR affected the colony forming potential of these cells. AML cell lines were first treated with 20-80 µM CUR, siRNA and/or combination of CUR and siRNA for 24 hours followed by analysis using methylcellulose colony assays. Figure 3 shows that AML colony-forming units (CFUs) were dramatically reduced by CUR and siRNA treatment alone but combination of CUR and siRNA have similar effect with CUR 40 µM alone.

**LSCs might be vetoed the inhibitory effects of OPN siRNA on colony forming potential via OPN overexpression towards CUR treatment**

**Figure 4:**
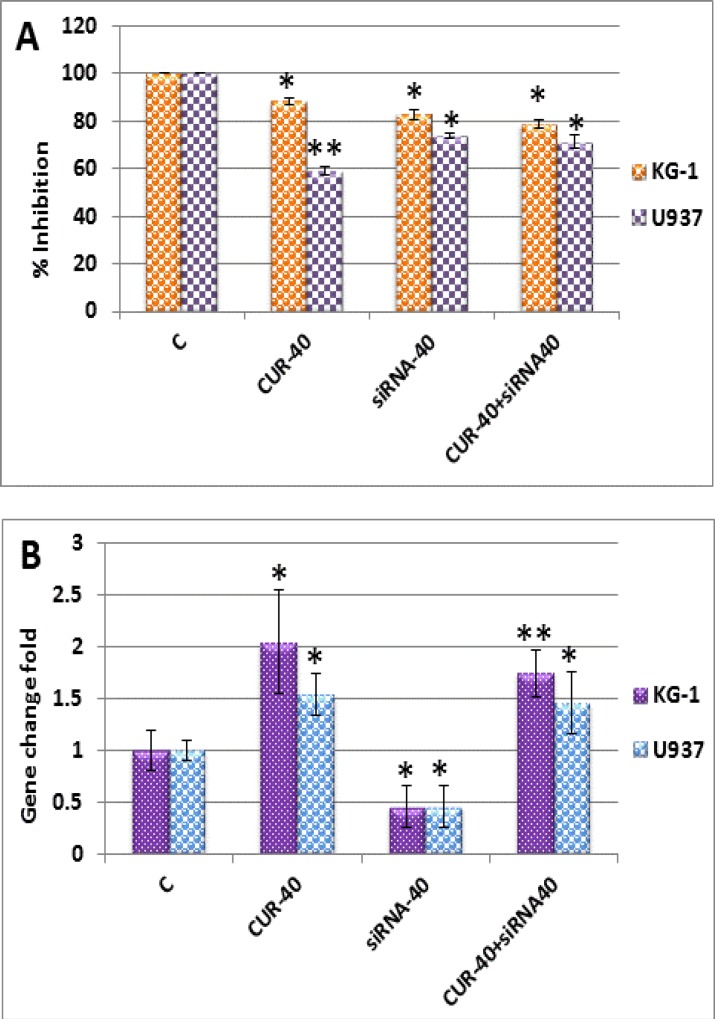
Result of osteopontin gene transfection with siRNA (40 pM) and treatment with curcumin (40 µM) in KG-1 and U937 cell lines. **(A):** Viability was assessed by MTT after transfection in both cell lines. **(B):** Results of Real-time PCR after transfection with in both cell lines showed that osteopontin could preclude curcumin-induced apoptosis and has additive effect with siRNA. Data are mean ± S.D. of three similar experiments.

To directly examine whether change in OPN gene expression, plays role in nullifying of inhibitory potency of CUR and siRNA on AML cells colonization. OPN expression was suppressed by siRNA and also the effect of OPN knockdown on in vitro proliferation was examined by MTT assay; then, results in mRNA levels were confirmed by Real-time PCR (Figure 4: A-B). Suppression of OPN expression by siRNA alone increased the susceptibility of these cell lines to apoptosis (17 ± 2 in KG-1 cells vs. 26 ± 1% in U937 cells), compared to combination of curcumin with siRNA (21 ± 1.7% in KG-1 cells vs. 29 ± 2.8% in U937 cells) (Figure 4: A-B).

These results suggest that CUR with enhancement of OPN gene in mRNA level could preclude CUR induced apoptosis and nullify the colony forming potential of OPN specific inhibitor in AML cell lines.

**Effect of curcumin on CD34+/CD38- isolated AML patient cells**

**Figure 5:**
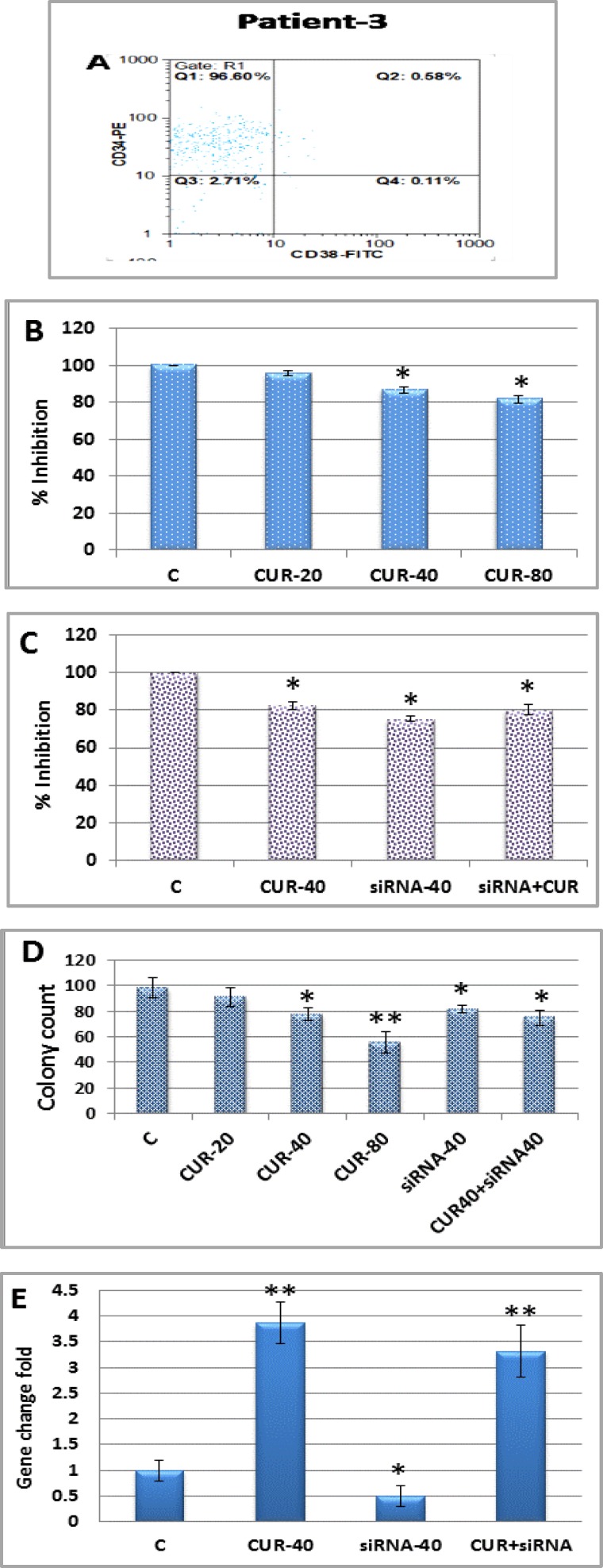
Curcumin was effective against primary CD34+/CD38- AML cells. **(A):** Primary CD34+/CD38- cells isolated from BMMCs of 10 AML patients and subjected to flow cytometry to determine the purity of CD34+/CD38- cells. **(B):** MTT assay were performed for all isolated AML samples which treated with different concentrations of curcumin (0, 20, 40 and 80 μM) for 24h. **(C):** Effects of siRNA (40 pM) transfection and curcumin treatment on growth inhibitory were examined by MTT and **(D):** Colony assay. Result on OPN gene expression **(E):** was evaluated by Real-time PCR. Values are given as mean ± SD of three independent experiments.

The cytotoxic effects of CUR on primary CD34+/CD38- as main phenotypes of AML LSCs were also examined. CD34+/CD38- cells were isolated from BMNCs of 10 AML patients by MACS Technology. The purity of isolated samples was more than 92% in most cases (Figure 5: A). CD34+/CD38- cells were treated with CUR (20, 40, 80 μM) for 24h and growth inhibitory effects were determined by MTT assay (Figure 5: B).

In order to verify the role of OPN gene expression in LSCs colonization, OPN gene were silenced in all AML samples with siRNA followed by CUR treatment for 24h.

**Figure 6:**
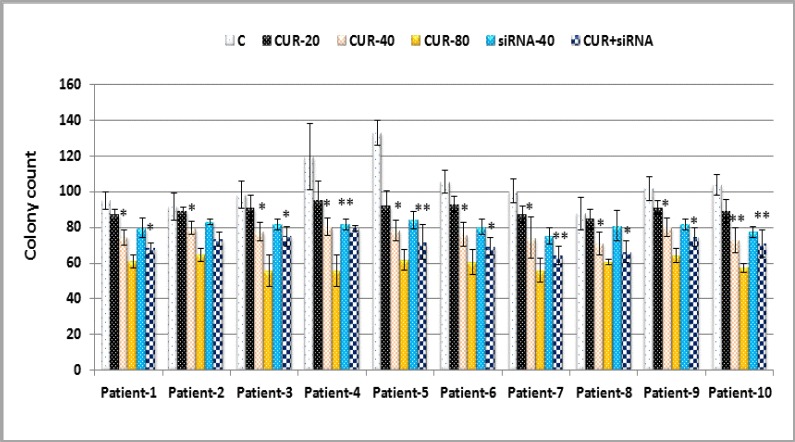
Effects of curcumin and siRNA on colony forming potential of primary CD34+/CD38- AML sample. Data are mean ± S.D. of three independent experiments. Statistical significance was defined at *p<0.05, **p<0.01 and ***p<0.001 compared to corresponding control

The effects of OPN knockdown on growth inhibitory were evaluated by MTT (Figure 5: C), colony assay (Figure 5: D and Figure 6) and in mRNA level assessed by Real-time PCR (Figure 5: E). Result showed that CUR inhibited proliferation and colonization potency of CD34+/CD38- LSCs depend on French-American-British (FAB) classifications, underlying cytogenetic and molecular changes. LSCs vetoed this effect via OPN overexpression.

**Table 2:** Nucleotide sequences of primers used for Real-time RT-PCR

**Gene**	**Accession Number**	**Forward Primer**	**Reverse Primer**	**Size (bp)**
HPRT	NM_000194	TGGACAGGACTGAACGTCTTG	CCAGCAGGTCAGCAAAGAATTTA	111
OPN	NM_001251830	ACCCTTCCAAGTAAGTCCAACG	GGTGAGAATCATCAGTGTCATCTAC	139

**OPN:** Osteopontin, **HPRT:** Hypoxanthine-guanine phosphoribosyl transferase

**Table 3:** SiRNA sequence

**Name**	**Sequence**
Sense (5’-3’)	GGAAUAUUACUGUGGGAAAdTdT
Antisense (5’-3’)	UUUCCCACAGUAAUAUUCCdTdT

## Discussion

 In contrast to well-defined association between OPN and solid tumor progression and metastasis,^27^^,^^29^ there is paucity of research on OPN in leukemia. OPN is an important component of Hematopoietic Stem Cell (HSC) niche which participates in HSC location and as a physiologic-negative regulator of HSC proliferation.^        9 ^ Hence, deregulated expression of OPN may play an important pathogenic role in hematological malignancies.

Targeting OPN and OPN-related signaling pathways may represent an important avenue for the development of therapeutics strategy.^        27 ^ The results of present study demonstrated that CUR induced apoptosis and reduced colonization potency in both U937 cells and KG-1 cells, as well as in primary CD34+/CD38- AML cells.

Curcumin inhibited cell growth and also induced apoptosis in both cell lines and patients primary cell culture. The residual cells (CD34+ 38- as main phenotype of LSCs) remained after treatment with CUR has evolved to produce an increased level of OPN expression. This is evidenced by co-treatment of CUR and siRNA did not show any synergic effect on reduction of colonization potency in AML cells. These data provide the first proof of principle that how OPN can maintain LSCs dormancy towards CUR treatment. Our Real-time analyses demonstrated that the expression of OPN was up-regulated by CUR treatment in both AML cell lines and primary AML cells.

We hypothesized that the acquired over expression of OPN in survival signaling pathway may interpret acquired resistance of LSCs towards CUR induced apoptosis. Since OPN plays an important role in the mechanism underlying drug resistance to CUR in AML cells, these data may provide evidence that how LSC is able to attenuate CUR induced apoptosis in AML.

In present study, we demonstrated that CUR could enhance apoptosis and decrease colony count in both KG-1 and U937 cell lines, as well as in primary CD34+/CD38- AML cells. In line with our result, Rao and colleagues reported that CUR significantly inhibited proliferation and clonogenic growth in a dose-dependent manner in CD34+ AML cells.^        30 ^

CD44 is one of OPN receptors and also functions as an anti-apoptotic protein. ^31^^,^^32^  CD44 is an important determinant of tumor progression and metastasis in various human malignant tumors and recently, its role was demonstrated in tumor stem cell biology.^33^^,^^34^ 

In good agreement with our result, Subramaniam et al. in their research on HT-29 as a human colon cancer cell line which produce a large amount of CD44 protein showed that after CD44 silencing with siRNA, cell lines were less adhesive to hyaluronan and more susceptible to apoptosis induced by etoposide.^35^

They claimed that siRNA CD44 clones formed a lower number and size of colonies in soft agar assays .On the other hand, siRNA CD44 cell clone xenografted in nude mice generated tumors with a reduced tumor volume and wet weight, as compared to control vector clone.

## CONCLUSION

 Our results suggested that the knockdown of OPN inhibited apoptosis in LSCs. Also, soft agar colony assays revealed that silencing of OPN using siRNA against OPN variants and partial cds significantly decreased colony numbers in both AML cell lines and primary CD34+/CD38- AML cells. Furthermore, LSCs with promotion of OPN overexpression towards CUR treatment might be nullify the inhibitory effects of OPN siRNA on colony forming potential and also maintain their survival.
